# The Involvement of Glial Cells in Blood–Brain Barrier Damage in Neuroimmune Diseases

**DOI:** 10.3390/ijms252212323

**Published:** 2024-11-17

**Authors:** Satoshi Nagata, Ryo Yamasaki

**Affiliations:** 1 Department of Neurology, Neurological Institute, Graduate School of Medical Sciences, Kyushu University, Fukuoka 812-8582, Japan; 2 Clinical Education Center, Kyushu University Hospital, Fukuoka 812-8582, Japan

**Keywords:** blood–brain barrier, endothelial cell, astrocyte, microglia, pericyte, multiple sclerosis, cytokine, chemokine

## Abstract

The blood–brain barrier and glial cells, particularly astrocytes, interact with each other in neuroimmune diseases. In the inflammatory environment typical of these diseases, alterations in vascular endothelial cell surface molecules and weakened cell connections allow immune cells and autoantibodies to enter the central nervous system. Glial cells influence the adhesion of endothelial cells by changing their morphology and releasing various signaling molecules. Multiple sclerosis has been the most studied disease in relation to vascular endothelial and glial cell interactions, but these cells also significantly affect the onset and severity of other neuroimmune conditions, including demyelinating and inflammatory diseases. In this context, we present an overview of these interactions and highlight how they vary across different neuroimmune diseases.

## 1. Introduction

The blood–brain barrier (BBB) is a complex network that protects the central nervous system (CNS) from invasion by toxic substances and immune cells. It is composed of vascular endothelial cells, the endfeet of astrocytes that are in contact with these cells, and pericytes [1]. Along with the endfeet, about one-third of astrocyte cell bodies are in direct contact with blood vessels [2]. Water exchange between endfeet and endothelial cells occurs via aquaporin 4 channels [3]. Vessels expressing tight junctions (TJs) that do not make contact with glial cells have been found to exhibit high vascular permeability [4]. Furthermore, studies using two-photon laser scanning microscopy reported that the removal of astrocytic endfeet covering blood vessels did not directly impact the BBB, with no extravascular leakage of Evans blue or dextran-bound fluorescein [5]. The loss of contact with astrocyte endfeet in an in vivo mouse model doubled the vessel size [6], and partial endfeet disruption by the local ablation of astrocytes in mice resulted in replacement by endfeet from nearby astrocytes and vascular endothelial cells covered by them [7]. In addition, damage to vascular endothelial cells altered mitochondrial dynamics in astrocyte endfeet and promoted BBB repair [8]. In large vessels, a perivascular lumen filled with cerebrospinal fluid (CSF) exists between the endfeet and endothelial cells [9]. Furthermore, a lack of the autophagy-related 7 gene in vascular endothelium downregulated the expression of fibronectin, a major vascular basement membrane (BM) component, and significantly reduced the coverage of astrocytes along brain microvessels [10]. The involvement of the BM in maintaining endothelial cell and astrocyte functionality has also been reported. In this article, we will summarize the relationship between the BBB and glial cells, specifically astrocytes, as well as the changes and interactions that occur between the BBB and glial cells in neuroimmune diseases. It is known that glial cells, including astrocytes and endothelial cells, have various interactions. Here, we provide an overview of these interactions and describe how they differ between neuroimmune diseases.

## 2. Glial Cells in Blood–Brain Barrier Damage

### 2.1. Astrocytes and the Blood–Brain Barrier

**Figure 1:**
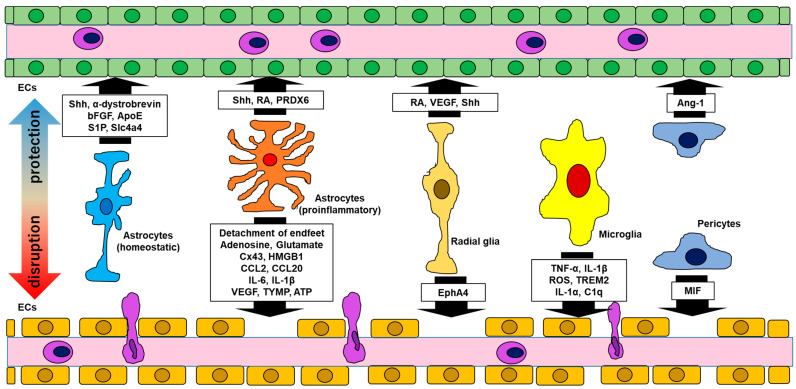
Interactions between the BBB and glial cells. Glial cells have either protective or disruptive effects, or both, on the BBB. Abbreviations: bFGF, basic fibroblast growth factor; ApoE, apolipoprotein E; S1P, sphingosine 1-phosphate; Slc4a4, solute carrier family 4 member 4; CCL, CC chemokine ligand; IL, interleukin; Shh, sonic hedgehog; RA, retinoic acid; PRDX, peroxiredoxin; Cx, connexin; HMGB, high mobility group box; VEGF, vascular endothelial growth factor; TYMP, thymidine phosphorylase; ATP, adenosine triphosphate; EphA4, ephrin A4; TNF, tumor necrosis factor; ROS, reactive oxygen species; TREM, triggering receptor of myeloid cells; Ang, angiotensin; MIF, migration inhibitory factor; ECs, endothelial cells.

Astrocytes exert their effects on the BBB through the production of a variety of bioactive substances. By producing sonic hedgehog (Shh), α-dystrobrevin, basic fibroblast growth factor, and apolipoprotein E, astrocytes alter the expression of tight junction proteins and affect vascular endothelial connectivity [4,11,12,13]. They also secrete sphingosine 1-phosphate (S1P), which stabilizes the vascular endothelial cytoskeleton and strengthens TJs [14]. The astrocyte-specific pH regulator solute carrier family 4 member 4 (Slc4a4) is involved in the maintenance of the BBB through its involvement in the CC chemokine ligand 2 (CCL2)/CC chemokine receptor 2 (CCR2) pathway, including the reduction in astrocyte CCL2 secretion [15] (Table 1, Figure 1). Studies with dentin matrix protein 1 (Dmp1)-expressing astrocytes reported the mechanisms regulating BBB integrity involved the transfer of mitochondria to endothelial cells via endfeet and the regulation of laminin and zonula occludens-1 (ZO-1) expression via aquaporin 4 expression [16,17]. The removal of perivascular astroglia by megalencephalic leukoencephalopathy with subcortical cysts 1 (Mlc1) gene-specific Cre, which is expressed specifically in perivascular astrocytes, promoted the abnormal localization of Claudin 5 and VE-Cadherin at the endothelial cell junction and microgliosis, suggesting that BBB defects associated with astroglial damage are involved in the development of neuroinflammation [18]. In addition, knockout of the Itga7 gene in astrocytes decreased the expression of laminin protein at the BM and decreased expression of claudin-5 and ZO-1 at TJs, as well as the adhesion of the α7 integrin subunit of astrocytes to laminin, resulting in reduced BBB integrity [19]. Connexins (Cx) are transmembrane proteins that form gap junction (GJ) channels that allow for the intercellular exchange of ions and energy sources [20,21], and in the CNS, they are expressed primarily in glial cells. Astroglia express Cx26, Cx30, and Cx43 [22,23,24,25,26]. Cx in astrocytes are not directly connected to the vascular endothelium, although they have a role in maintaining brain metabolic homeostasis [27,28].

### 2.2. Radial Glial Cells and the Blood–Brain Barrier

Radial glial cells (RGs), which are considered the main source of astrocyte progenitor cells, were reported to interact extensively with vascular endothelial cells [74]. RGs express retinoic acid (RA) and vascular endothelial growth factor (VEGF), which interact with RA receptors and VEGF receptor 2 on endothelial cells, respectively [52,53,54,75]. Furthermore, the removal of RGs reduced vascular density and branching, and the mechanism for this might involve the stabilization of vessels by inhibiting Wnt signaling, which controls the expression of matrix metalloproteinase (MMP) involved in BM destruction [76]. In addition to astrocytes, RG have also been shown to express Shh [55]. Furthermore, RGs express Ephrin A4 (EphA4) [56], which promotes vascular endothelial cell dysfunction [57,77] (Table 1, Figure 1).

### 2.3. Reactive Astrocytes and Blood–Brain Barrier Protection

Activated astrocytes have been reported to protect and disrupt the BBB. Reactive astrocytes release Shh, which increase lesion Shh levels further via a positive feedback loop [4,29,30]. The signaling inhibition of Shh was reported to enhance paracellular or transcellular cell migration [4,78], but studies using models with inactivated Shh signaling pathways reported that only transcellular cell migration was hampered [78]. In addition, reactive astrocytes increased the level of RA production, thereby increasing BBB integrity, and Shh directly affected inflammatory immune cells and had anti-inflammatory effects on neuroinflammation by inhibiting cell migration to and beyond the BBB [4,31] (Table 1, Figure 1).

### 2.4. Reactive Astrocytes and Blood–Brain Barrier Damage

Many previous studies have shown that reactive astrocytes compromise BBB integrity. Interleukin (IL)-1β produced by reactive astrocytes suppressed Shh production and promoted the release of inflammatory chemokines from astrocytes [47,79]. Furthermore, IL-1β upregulated ICAM-1 expression in endothelial cells and promoted the transcellular BBB migration of immune cells [48,80]. IL-6 secreted by reactive astrocytes reduced BBB function [45] and promoted T cell infiltration into the CNS [81]. Reactive astrocyte-derived VEGF and thymidine phosphorylase (TYMP) suppressed claudin-5 and occludin and promoted CD4+ T cell migration [49,50]. VEGF also regulated adhesion factors to enhance BBB permeability and promote immune cell migration by upregulating ephrin [82] (Table 1, Figure 1). In experimental autoimmune encephalomyelitis (EAE), a mouse model of MS, the expression of Cx43 by astrocytes was markedly reduced in demyelinating lesions during the acute phase of disease, indicating that Cx43 deficiency promotes leukocyte migration via the activation of endothelial cells [35]. However, Une et al. reported that the Cx43-specific knockout of astrocytes suppressed glial inflammation, reduced infiltrating immune cells, and ameliorated the severity of EAE [36]; therefore, it is important to be cautious when considering whether Cx43 deficiency has a proinflammatory effect on inflammatory cell infiltration.

### 2.5. Microglia and the Blood–Brain Barrier

Most previous reports on the interaction between glial cells and BBB have studied astrocytes, as described above; however, microglia also affect vascular endothelial cells. Imaging studies of living mice showed that astrocytes and microglia are in contact with vascular endothelial cells and interact with them, especially the endfeet-free regions of astrocytes [83]. Studies using imaging techniques such as confocal laser scanning microscopy and electron microscopy have shown that microglial projections cover approximately 15% of the endothelial cell surface [84]. Microglia affect the expression of BBB tight junction proteins, and tumor necrosis factor (TNF)-α and IL-1β released from microglia reduced the expression of occludin, ZO-1, and other proteins, increasing the BBB permeability [58,59] (Table 1, Figure 1). Furthermore, TNF-α and IL-1β act on immune cells to promote paracellular and transcellular migration beyond the BBB, respectively [80]. Furthermore, the binding of purines released from pannexin 1 (PANX1) channels on vascular endothelial cells to P2RY12 receptors on microglia was associated with vasodilation and increased blood flow [85], and blocking P2RY12 receptors on microglia decreased the vascular endothelial connectivity and reduced the cerebral blood flow [84]. In addition, microglia regulated the cerebral blood flow via the activation of C-X3-C motif chemokine receptor 1 (CX3CR1) and the production of reactive oxygen species (ROS) [86,87]. ROS are produced from activated microglia through the stimulation of nicotinamide adenine dinucleotide phosphate (NADPH) oxidase. In addition, ROS increases BBB permeability by decreasing the expression of VE-cadherin, occludin, and claudin-5 on the BBB [46,60,61]. The secretion of IL-1β from activated microglia triggered the release of VEGF and TYMP from reactive astrocytes and promoted the migration of immune cells to the CNS [49,88]. Moreover, IL-1α, TNF-α, and C1q released from activated microglia indirectly affected the BBB by inducing reactive astrocytes [62] (Table 1, Figure 1).

### 2.6. Pericytes and the Blood–Brain Barrier

Pericytes reside between endothelial cells and astrocytes, and especially within the BBB, an endothelial basement membrane exists between pericytes and ECs [89,90]. Furthermore, pericytes interact with astrocytes and influence the distribution of AQP4 channels [91]. A subset of pericytes has the same origin as macrophages [92], and pericytes derived from mouse models of Alzheimer’s, epilepsy, and stroke and from human brains with these diseases were reported to have acquired a microglial phenotype [93,94,95]. The impact of pericytes on the BBB is related to the maintenance of TJs and regulation of transcellular migration through the BBB [89,96,97]. Angiotensin-1 released from pericytes binds to Tie-2, a receptor expressed on ECs, leading to vascular stability and maturation [64]. Pericytes respond to inflammatory mediators such as TNF-α in an inflammatory environment, which increases ICAM-1 expression on endothelial cells and releases macrophage migration inhibitory factor (MIF) [65]. MIF was recently reported to promote the apoptosis of vascular endothelial cells, leading to the exacerbation of inflammation [66] (Table 1, Figure 1).

## 3. The Involvement of Glial Cells in Blood–Brain Barrier Damage in Demyelinating Diseases

### 3.1. Glial Cells in Blood–Brain Barrier Damage in Multiple Sclerosis

#### 3.1.1. Pathogenic Cell Migration in Multiple Sclerosis

In inflammatory diseases, represented by multiple sclerosis (MS), pathogenic immune cells invade the CNS through the BBB to cause the disease. The steps of leukocyte migration begin with rolling, followed by adhesion, clawing, and permeabilization. In the rolling step, leukocytes adhere to the vascular endothelial surface through various selectin-mediated interactions with vascular endothelial cells. They then migrate against the blood flow, which is called clawing, to find areas where they can migrate through the BBB [98]. The role of cell adhesion molecules (CAMs) is central to transcellular T cell migration, which typically involves interactions between intercellular adhesion molecule-1 (ICAM-1) or vascular cell adhesion molecule-1 (VCAM-1) expressed on endothelial cells and lymphocyte function-associated antigen-1 (LFA-1) or very late antigen-4 (VLA-4) expressed on T cells [99]. Platelet endothelial cell adhesion molecule-1 (PECAM-1) expression on endothelial cells is concentrated at intercellular junctions, where it binds to αvβ3 integrin on leukocytes and is involved in cell migration. It also binds to itself to connect endothelial cells. In MS patients, elevated levels of PECAM-1 in the serum and CSF have been reported [100,101]. Other CAMs include activated leukocyte cell adhesion molecule (ALCAM), melanoma cell adhesion molecule (MCAM), and domain-containing cell adhesion molecule (DICAM). DICAM promotes inflammation-induced CD4+ Th17 cell migration and is upregulated in patients with active relapsing–remitting and progressive MS. In addition, monoclonal antibodies targeting DICAM improved EAE [102]. Molecular magnetic resonance imaging (MRI) and in vitro analysis showed that MCAM was upregulated in endothelial cells in MS lesions and that MCAM promoted the migration of Th1 and Th17 cells [103]. DICAM was initially associated with BBB-related astrocytes in the CNS [104], but in MS patients, an increased frequency of DICAM+ CD4+ T cells in the peripheral blood and increased expression of its ligands, DICAM and αvβ3 integrin, were observed on endothelial cells in CNS lesions [102]. Following BBB permeabilization, pathogenic cells migrate into the CNS in response to a concentration gradient of chemokines on the abluminal side.

Recent studies have reported that cytokines and chemokines released from glial cells are also associated with BBB damage [105]. CCL2 is a cytokine that promotes BBB destruction [41], and S1P receptor agonists and the anti-rheumatic drug iguratimod inhibit CCL2 released from astroglia and stabilize the BBB [42,106] (Table 1, Figure 1). Leukocytes pass through the endothelial basement membrane to penetrate the perivascular space. To enter the brain parenchyma, cells must pass through the glia limitans and associated basement membrane, where matrix metalloproteinases (MMPs) produced from microglia disrupt these membranes and facilitate leukocyte migration into the CNS [107]. Elastin-derived peptides (EDPs), which are produced by the breakdown of elastin under an inflammatory environment, were reported to suppress the expression of MMPs from astrocytes by suppressing the peroxisome proliferator-activated receptor gamma (PPARγ) in cultured astrocytes. Furthermore, EDPs are thought to be involved in the regeneration of CNS tissue [108,109,110].

#### 3.1.2. Reactive Astrocytes and the Blood–Brain Barrier in Multiple Sclerosis

In acute and chronic MS lesions, reactive astrocytes induce demyelination [111] and, as noted above, they have increased RA production, which tightens the BBB. Studies using the EAE model reported that Shh directly affected pathogenic immune cells and protected against neuroinflammation by inhibiting BBB permeabilization [4,31]. Mild EAE severity was reported in a mouse model with an activated Shh signaling pathway in astrocytes [112]. The expression of the antioxidant protein peroxiredoxin 6 (PRDX6) was increased in the spinal cord of EAE mice, and PRDX6 inhibited BBB destruction [32] (Table 1, Figure 1). Furthermore, IL-1β produced by reactive astrocytes suppressed astrocyte Shh production and promoted the release of inflammatory chemokines from astrocytes [47,79]. In addition, the high expression of ICAM-1 in endothelial cells promoted the transcellular BBB migration of immune cells [48,80]. Recently, the bioactive chalcone compound isoliquiritigenin was reported to inhibit reactive astrocytes and reduce the levels of cytokines in the CNS, including those of IL-1β [113]. VEGF and TYMP derived from reactive astrocytes in MS and other neuroinflammatory diseases suppressed claudin-5 and occludin and promoted CD4+ T cell migration [49,50]. Moreover, IL-6 produced by reactive astrocytes reduced the barrier function of the BBB [45]. Recently, taurochenodeoxycholic acid was shown to inhibit the activation of astrocytes in EAE mice and suppress IL-6 and other cytokines, which is expected to protect the BBB [114]. In MS patients and animal models, there are also changes in cell-to-cell contact, including the detachment of astrocytic endfeet from endothelial cells [33,34]. Recent studies reported that astrocytes in EAE expressed high levels of high mobility group box 1 (HMGB1), a non-histone DNA-binding nuclear protein involved in MS, and that the knockout of HMGB1 in astrocytes in EAE mice enhanced claudin-5 expression and decreased ICAM1 and VCAM1 expression [40] (Table 1, Figure 1).

Connexin (Cx) is a component of gap junctions, which connect the cytoplasm between cells. Previous studies have reported the loss of astroglial Cx30 and Cx43 in acute MS lesions and significant upregulation of Cx43, reflecting astrogliosis in chronic lesions [115,116]. The loss of Cx43 promotes leukocyte migration via the activation of vascular endothelial cells and is associated with progressive MS [35,115]. In contrast, as mentioned above, Cx43-specific knockout in astrocytes has been reported to suppress glial inflammation, reduce infiltrating immune cells, and decrease the severity of EAE [36]. Increased Cx43 in the chronic phase may reflect Cx hemichannels on astrocytes, and blocking Cx43 hemichannels improved the severity of disease in the chronic phase of EAE [117]. Furthermore, astrocytes with increased Cx43 hemichannel activity showed increased levels of adenosine triphosphate (ATP), glutamate, adenosine, and inflammatory substances, resulting in neuronal damage [37,51]. In particular, glutamate and adenosine have been reported to increase the permeability of the BBB [38,39] (Table 1, Figure 1). Damage to the BBB by the Cx43 hemichannel may be involved in MS relapses and exacerbations in chronic MS.

#### 3.1.3. Microglia and the Blood–Brain Barrier in Multiple Sclerosis

TNF-α and IL-1β released from activated microglia found in MS reduced the expression of occludin and ZO-1, and increased the permeability of the BBB [58,59] (Table 1, Figure 1). Furthermore, TNF-α and IL-1β affected immune cells by promoting paracellular and transcellular migration through the BBB, respectively [80]. A recent study reported that ginsenoside, the main active ingredient of ginseng, and Icariin, a natural flavonoid compound, as well as high-density lipoprotein, suppressed the expression of IL-1β and other inflammatory cytokines by inhibiting microglial activation and promoting BBB integrity in EAE mice [118,119,120]. Activated microglia-derived ROS are produced via the activation of NADPH oxidase. Furthermore, ROS enhances BBB permeability by decreasing the expression of VE-cadherin, occludin, and claudin-5 on the BBB [46,60,61]. Dabrafenib was found to suppress ROS production in cultured microglia by inhibiting the cell cycle, and it has been reported as a new therapeutic candidate for MS [121]. The secretion of IL-1β from activated microglia triggers the release of VEGF and TYMP from astrocytes and promotes the migration of immune cells into the CNS [49,88]. IL-1 α, TNF-α, and C1q released from activated microglia also indirectly affect the BBB by inducing A1 astrocytes, which are a cytotoxic type of reactive astrocyte [62] (Table 1, Figure 1). Recently, the bioactive compound Astragalus polysaccharide was reported to suppress IL-1α, TNF-α, and C1q production in an in vitro microglia–astrocyte co-culture model by suppressing Sema4D-PlexinB2 signaling, followed by the inhibition of microglia and astrocyte activation [122].

#### 3.1.4. Disease-Modifying Drugs and the Blood–Brain Barrier in Multiple Sclerosis

Current disease-modifying drug (DMD) therapies for MS include IFNβ and natalizumab, which inhibit relapsing MS through their effects on the BBB. IFNβ improves BBB damage and integrity in relapsing–remitting multiple sclerosis patients, and reduces the endothelial permeation of inflammation-induced CD4+ Th1 cells [123]. Natalizumab, a monoclonal antibody against α4β1 integrin, the ligand for VCAM-1, directly inhibits the BBB permeabilization of immune cells, reducing further BBB destruction and inflammation [124]. Natalizumab is associated with a risk of progressive multifocal leukoencephalopathy (PML), in which the reactivation of the JC virus (JCV) causes progressive demyelination within the CNS [125,126]. The proportion of PML patients among natalizumab users is reported to be 4.3%, the highest figure for DMD overall compared with other drugs, including rituximab 2.9%, fingolimod 0.53%, and dimethyl fumarate 0.20% [127]. Clinical manifestations in early PML include a variety of neurological symptoms, including cognitive dysfunction, visual field disturbances, seizures, and movement disorders. JCV infection is caused by person-to-person contact or the oral ingestion of contaminated food or water [128,129]. Natalizumab inhibits CD4+ T cell migration to the CNS, and thus, the depletion of CD4+ T cells necessary for JCV elimination from the CNS is thought to be one of the causes of disease onset [130]. Recent reports have shown that MCAM+CCR6+Th17 cells gradually acquire a pathogenic profile during natalizumab treatment, involving inflammation-induced cytokine production, leading to BBB damage and oligodendrocyte damage, accompanied by increased Th17 cell frequency in the cerebrospinal fluid of natalizumab-treated patients. The possibility that MCAM+CCR6+Th17 cells are involved in the pathophysiology of rebound after natalizumab discontinuation has been raised [131]. Furthermore, S1P receptor agonists such as fingolimod and siponimod have also been reported to affect the BBB. S1P1 receptors are responsible for T cell efflux from lymph nodes, and S1P receptor agonists were reported to bind to and downregulate S1P1 receptors on the surface of lymphocytes, thereby inhibiting T cell efflux from lymph nodes and reducing the number of circulating autoreactive T cells [132]. S1P receptors expressed on vascular endothelial cells and astrocytes were reported to decrease the permeability of endothelial cells in an in vitro model [133] (Table 1). In addition, the permeability-decreasing effect on the BBB and the reduction in endothelial cell damage by S1P receptor agonists through astrocytes have also been reported, which were related to the reduction in peripheral blood mononuclear cell (PBMC) migration by the direct effects of S1P receptor agonists on endothelial cells and astrocytes in co-culture [42,134].

### 3.2. Glial Cells in Blood–Brain Barrier Damage in Neuromyelitis Optica Spectrum Disorders

Neuromyelitis optica spectrum disorders (NMOSDs) are caused by the destruction of astrocytes through complement-dependent cytotoxicity promoted by autoantibodies against aquaporin4 (AQP4) channels in astrocyte endfeet [135,136,137]. In particular, the destruction of astrocyte endfeet was reported to induce the cellular infiltration of lymphocytes and other immune cells [138]. However, the pathogenesis of NMOSDs requires the influx of anti-AQP4 antibodies produced by peripheral blood plasma cells into the CNS beyond the BBB, and the mechanism involved in this is still largely unknown. Compared with MS, there are limited reports on BBB alterations in NMOSDs.

The following mechanisms have been reported before the onset of disease: IL-6, TYMP1, and MMP2 are elevated in the spinal fluid of NMOSD patients, and the increase in MMP2 induced by IL-6 production via the NF-κB signaling pathway destroys the BBB, allowing serum anti-AQP4 antibodies to migrate into the CNS [139,140]. Furthermore, exposure to humoral factors in the serum of NMOSD patients produces MMP2 and MMP9 from vascular endothelial cells, increasing the BBB permeability and further increasing VCAM expression [67] (Table 1). Anti-AQP4 antibodies isolated from NMOSD patients increase the in vitro production of inflammatory cytokines such as interferon-inducible protein 10 (IP-10), IL-6, IL-1β, and CXCL3 from microcerebral vessels isolated from rat brain, and also decrease the expressions of Claudin5 and other TJ proteins [69,141]. CCL2 is thought to be involved in the development of NMOSDs because NMOSD patients have markedly elevated CCL2 levels in the spinal fluid; furthermore, the inhibition of astrocyte CCL2 expression has been found to ameliorate anti-AQP4 antibody-induced neuronal damage in vivo and in vitro, and CCL2 itself can destroy the BBB [41,141] (Table 1). Furthermore, regarding the mechanism of anti-AQP4 antibodies invading the CNS, GRP78 autoantibodies that target endothelial cells were reported to allow the passage of AQP4 antibodies through the BBB and contribute to the onset and severity of NMOSDs [142,143]. Cx was reported to be associated with NMOSDs and IgG from NMOSD patients disrupted gap junctions and increased connexin hemichannels on cultured astrocytes. Astrocytes with elevated Cx43 hemichannel activity release ATP, glutamate, adenosine, and inflammatory substances, leading to progressive neuronal damage [37,51]. Glutamate and adenosine were reported to increase the permeability of the BBB [38,39] (Table 1, Figure 1) and BBB disruption caused by Cx43 hemichannels might contribute to the development of NMOSDs.

## 4. Involvement of Glial Cells in Blood–Brain Barrier Damage in Other Neuroimmune Diseases

### 4.1. Glial Cells in Blood–Brain Barrier Damage in Anti-NMDA Receptor Antibody Encephalopathy

Anti-N-methyl-D-aspartate receptor (NMDAR) encephalitis is the most common antibody-associated autoimmune disease, accounting for approximately 81% of autoimmune encephalitis cases, and it is more common in young women presenting with acute psychiatric symptoms and seizures [144,145,146,147]. In the CNS, NMDARs are expressed mainly in neurons in the prefrontal cortex, hippocampus, amygdala, and hypothalamus [148], and pathogenic anti-NMDAR antibodies internalize NMDARs on the cell surface, reducing the density of NMDARs at the synapse [149]. Anti-NMDAR antibodies are produced by B cells derived from the periphery [150,151] and anti-NMDAR antibodies administered to the peripheral blood only caused symptoms in mice with an impaired BBB [152], suggesting that disease onset requires an influx of antibodies into the CNS via a disrupted BBB [153]. Although the relationship between NMDARs and the BBB remains unclear, an association between encephalitis severity and the degree of BBB damage was reported when the ratio of albumin in the CSF to that in the serum was used in patients with anti-NMDAR encephalitis [154]. NMDARs, when activated, can destroy the BBB through the increased expression of MMP2 and MMP9, or alter its permeability by changing the expression of TJs [68,155,156]. Recent reports indicate that NMDARs are also expressed on the BBB, and that NMDAR activation leads to BBB damage via the phosphatidylinositol 3-kinase (PI3K)/threonine kinase (Akt)/mammalian target of rapamycin (mTOR) signaling pathway [73]. Furthermore, the intraperitoneal administration of PBMCs from patients with NMDAR encephalitis causes BBB destruction [157]. The activation of the PI3K/Akt/mTOR pathway in a mouse model of anti-NMDAR encephalitis also reduced ZO-1 and Claudin-5 expression and increased BBB permeability [158] (Table 1). Although few studies have investigated an association between this disease and astrocytes and microglia, NMDARs are expressed on astrocytes in the cortex and hippocampus [159,160]. Anti-NMDAR antibodies that enter the CNS promoted the internalization of NMDARs on astrocytes and neurons, leading to the release of neuropathic factors such as ATP [161]. Regarding microglia, it was recently reported that increased triggering receptor of myeloid cells 2 (TREM2), CD44, and MMP9 levels in patient CSF reflected microglial activation. Especially, TREM2 was associated with the increased permeability of the BBB [63] (Table 1, Figure 1).

### 4.2. Glial Cells in Blood–Brain Barrier Damage in Anti-VGKC Antibody Encephalopathy

Encephalitis caused by anti-voltage-gated potassium channel (VGKC) complex antibodies resulted in limbic encephalitis, a disease characterized by memory impairment, psychiatric symptoms, and hyponatremia [162,163]. The pathological analysis of patients with limbic encephalitis demonstrated low-to-moderate T cell infiltration as well as gliosis and active microglia in the hippocampus, resulting in neurodegeneration, when compared with other encephalitis patients. In addition, immunoglobulin and complement C9neo were deposited on the surface of neurons. Therefore, complement activation is thought to be one of the key mechanisms of neurodegeneration [164]. In studies of animal models, VGKC antibodies were distributed throughout the brain and the disease pathology included disorders of the limbic system, including the hippocampus, which were partly related to the selective impairment of the BBB in various brain regions [165]. Other reports suggest that the concentration of IL-10 in the CSF from patients with limbic encephalitis was higher than in other autoimmune encephalopathies, including anti-NMDAR encephalitis, and that the anti-inflammatory effect of IL-10 suppressed the local inflammatory response [166]. Much is still unknown about the specific effects of anti-VGKC complex antibodies on the BBB and glial cells in encephalitis.

### 4.3. Glial Cells in Blood–Brain Barrier Damage in Neuropsychiatric Systemic Lupus Erythematosus

Systemic lupus erythematosus (SLE) is a systemic autoimmune inflammatory disease, and approximately 40–75% of SLE patients experience neuropsychiatric symptoms, including mood disorders, acute confusion, and cognitive dysfunction, called neuropsychiatric-SLE (NPSLE) [167]. Although the exact mechanism of NPSLE is unknown, a combination of inflammatory cytokines, autoantibodies, and BBB disorders are involved [168,169]. Anti-DNA antibodies and NMDAR cross-reactivity in SLE patients were reported to cause the neuropsychiatric symptoms [170], and as in NMDAR encephalitis, NPSLE developed in SLE when anti-DNA antibodies in the peripheral blood invaded the CNS associated with BBB damage [171]. Furthermore, it was suggested that the greater the damage to the BBB, the more severe the NPSLE symptoms may become [172]. Studies of mouse models of SLE reported increased BBB permeability and the activation of astrocytes and microglia [173]. Therefore, the involvement of the BBB and glial cells in SLE is significant. However, microglial activation occurred in the hippocampus before BBB damage occurred [174], and the results of future studies are expected.

### 4.4. Glial Cells in Blood–Brain Barrier Damage in Behçet’s Disease

Behçet’s disease (BD), a multisystem inflammatory disease of unknown cause, was first reported in 1937 associated with three signs of recurrent ulcers in the oral cavity or pubic region, and uveitis [175]. Neuro-BD (NBD) is defined as neurological symptoms in BD patients, with a variety of clinical manifestations, including brainstem syndrome, MS-like symptoms, movement disorders, and meningoencephalitis syndrome [176]. Although not many studies have investigated the relationship between NBD and BBB, the pathological analysis of autopsy brain sections from NBD patients revealed the infiltration of inflammatory cells, including T cells and monocytes, around small blood vessels with neuronal loss, especially in the brainstem [177]. Furthermore, a study examining immunoglobulin (Ig) profiles in the CSF of NBD patients showed that Ig was upregulated in NBD with BBB damage compared with NBD without damage. Therefore, Ig might pass through the damaged BBB [178].

### 4.5. Glial Cells in Blood–Brain Barrier Damage in Vasculitis

Other vasculitis disorders, excluding SLE and BD, include diseases that cause CNS involvement, such as granulomatosis polyangiitis (GPA), microscopic polyangiitis (MPA), and Takayasu arteritis. In the context of vasculitis in relation to vascular endothelial cells, it was reported that various autoantibodies against vascular endothelial cells, anti-endothelial cell antibodies (AECAs), are present, depending on the disease. Their pathogenic potential was indicated by the finding that AECAs can trigger inflammatory processes and induce the apoptosis of endothelial cells via complement-dependent or antibody-dependent cell injury [179]. In particular, in GPA, AECAs, together with antineutrophil cytoplasmic antibodies (ANCAs), activate endothelial cells to secrete IL-1β, IL-6, IL-8, and CCL2, which decreases BBB integrity and upregulates the expression of adhesion molecules, including E-selectin, ICAM-1, and VCAM-1 [70,71,72] (Table 1). In Takayasu arteritis, AECAs induce the production of IL-4, IL-6, and IL-8 from the vascular endothelium of the aorta [180]. Anti-heat shock protein (Hsp60) antibody, another AECA, was detected in patients with SLE, polyarteritis nodosa (PN), GPA, MPA, eosinophilic polyangiitis granulomatosa (EGPA), and BD. Anti-Hsp60 antibody has been shown to cause the apoptosis of vascular endothelial cells in culture [181]. Vascular endothelial proteins recognized as target antigens by IgG in the serum of patients with ANCA-associated vasculitis interact with transforming growth factor-β (TGF-β), which is associated with apoptosis [182]. Therefore, AECAs can induce apoptosis in vascular endothelial cells.

### 4.6. Glial Cells in Blood–Brain Barrier Damage in GFAP Astrocytopathy

The disease concept of autoimmune glial fibrillary acidic protein (GFAP) astrocytopathy was reported in 2016 [183]. This disease exhibits a variety of symptoms, mainly meningoencephalitis, and is characterized by the presence of GFAP-specific IgG in the CSF or serum [184]. This antibody is considered a biomarker, but not a pathogenic antibody, because it recognizes an intracellular antigen [185], and the pathogenic events leading to antibody production are based on infection, trauma, or the presence of autoimmune disease [43]. The relationship between this disease and BBB damage remains unclear, but characteristic MRI findings of this disease suggest radial linear perivascular and periventricular enhancement [184,186]. Furthermore, the histopathological analysis of soft membrane biopsies has revealed the infiltration of CD8+ lymphocytes and macrophages [185]. Brain biopsies have also shown inflammatory cell infiltrates around blood vessels and the activation of microglia [187], suggesting the presence of BBB damage in this disease. CCL20 is elevated in the CSF of patients, and although the source of the producing cells is unclear, activated astrocytes in GFAP astrocytopathy secrete CCL20, which promoted the migration of lymphocytes to the CNS and increased BBB permeability [43,44]. Elevated levels of TNF-α and IL-6 in the CSF of patients have also been reported. IL-6 decreased the in vitro BBB function [45] and TNF-α derived from M1-like microglia induced necroptosis in vascular endothelial cells [188], indicating that both cytokines may be associated with BBB damage in GFAP astrocytopathy.

## 5. Conclusions

This review focused on the relationship between the BBB and glial cells, represented by astrocytes, and on the changes in and interactions of the BBB and glial cells in neuroimmune diseases. In the inflammatory milieu generally seen in neuroimmune diseases, changes in the surface molecules of vascular endothelial cells, as well as changes in binding molecules and the weakening of cell-to-cell connections, facilitate the entry of inflammation-induced immune cells and disease-related autoantibodies into the CNS. Glial cells affect the binding strength of vascular endothelial cells by altering their own morphology and by releasing cytokines, chemokines, and bioactive molecules. Among neuroimmune diseases, the association between the disease and vascular endothelial cells or glial cells has been most frequently studied in MS. However, vascular endothelial cells and glial cells also have a significant impact on the onset and severity of other neuroimmune diseases such as demyelinating diseases and inflammatory diseases. In MS, vascular endothelial cells are one of the targets of therapeutic agents, and it is expected that novel therapeutic agents targeting vascular endothelial cells and glial cells will emerge in the future, not only for MS but also for other neuroimmune diseases.

## Figures and Tables

**Table 1:** Interactions between the BBB and glial cells.

	Functional Molecules	Impact on the BBB	Related Diseases	Reference
**Astrocytes (homeostatic)**	Shh α-dystrobrevin bFGF ApoE S1P Slc4a4	BBB integrity ↑ BBB integrity ↑ L-glucose permeability ↓ BBB stability BBB integrity ↑ BBB stability (related to CCL2 ↓)	- - - - - -	Alvarez 2011 [4] Lien 2012 [11] Sobue 1999 [12] Bell 2012 [13] Garcia 2001 [14] Ye 2024 [15]
**Astrocytes (proinflammatory)**	Shh ↑ RA ↑ PRDX6 ↑ ------------------------------------------ Detachment of endfeet Cx43 ↓(acute phase) Cx43 ↑, Adenosine ↑, Glutamate ↑ (chronic phase) HMGB1 ↑ CCL2 ↑ CCL20 ↑ IL-6 ↑ IL-1β ↑ VEGF ↑, TYMP ↑ ATP ↑	BBB integrity ↑ BBB protection (integration ↑) BBB protection --------------------------------------------------- BBB permeability ↑ BBB permeability ↑ or ↓ BBB permeability ↑ Claudin5 ↓, ICAM-1 ↑, VCAM-1 ↑ BBB integrity ↓ BBB integrity ↓ VE-cadherin ↓, occluding ↓, claudin5 ↓ (BBB integrity ↓) Shh ↓, ICAM-1 ↑ BBB integrity ↓ (claudin5 ↓, occluding ↓) BBB permeability ↑	MS MS MS MS MS, NMOSD MS, NMOSD MS MS, NMOSD GFAPA MS MS MS NMDARE	Alvarez 2011 [4], 2013 [29]; Sirko 2013 [30] Mizee 2014 [31] Yun 2015 [32] Eilam 2018 [33]; Prineas & Lee 2019 [34] Brand-Schieber 2005 [35]; Une 2021 [36] Yamasaki 2023 [37]; Bynoe 2015 [38]; Vazana 2016 [39] Shi 2022 [40] Xiao 2020 [41]; Spanpinato 2022 [42] Kimura 2019 [43]; Zheng 2023 [44] Chang 2015 [45]; Rochfort 2014 [46] Wang 2014 [47], Abadier 2015 [48] Argaw 2012 [49]; Chapouly 2015 [50] Bennett 2012 [51]; Yamaski 2023 [37]; Bynoe 2015 [38]
**Radial glia**	RA VEGF Shh ------------------------------------------ EphA4	BBB development BBB development BBB integrity↑ -------------------------------------------------- BBB dysfunction	- - - -	Mizee 2013 [52] Sentilhes 2010 [53]; Silva 2019 [54] Radonjic 2014 [55] Cheng 2002 [56]; Chen 2018 [57]
**Microglia**	TNF-α ↑, IL-1β ↑ ROS ↑ IL-1α ↑, TNF-α ↑, C1q ↑ TREM2↑	BBB integrity ↓ (ZO-1 ↓, occluding ↓) BBB integrity ↓ (VE-cadherin ↓, occludin↓, claudin5 ↓) (inducing proinflammatory astrocytes) BBB integrity ↓	MS MS MS NMDARE	Nishioku 2010 [58]; Shigemoto-Mogami 2018 [59] Sumi 2010 [60]; Rochfort 2014 [46]; Schreibelt 2007 [61] Liddelow 2017 [62] Chang 2023 [63]
**Pericytes**	Ang-1 ↑ MIF ↑	BBB stability ↑ Endothelial cell apoptosis ↑	- (Inflammation)	Gaengel 2009 [64] Stark 2013 [65]; Li 2023 [66]
**Endothelial cells**	MMP2/MMP9 ↑ IL-6 ↑ IL-1β ↑ IL-8 ↑ CCL2 ↑ NMDAR ↑	VCAM-1 ↑, BBB integrity ↓ VE-cadherin ↓, occluding ↓, claudin5 ↓ (BBB integrity ↓) Shh ↓, ICAM-1 ↑ BBB integrity ↓ BBB integrity ↓ BBB integrity ↓	NMOSD, NMDARE NMOSD, Vasculitis NMOSD, Vasculitis Vasculitis Vasculitis NMDARE	Tasaki 2014 [67]; Chen 2016 [68] Covo-Calvo 2020 [69]; Muller Kobold 1999 [70]; Del Papa 1996 [71] Del Papa 1996 [71] Del Papa 1996 [71]; Sun 2016 [72] Del Papa 1996 [71] Huang 2024 [73]

Abbreviations: ↑, increase; ↓, decrease; BBB, blood–brain barrier; bFGF, basic fibroblast growth factor; ApoE, apolipoprotein E; S1P, sphingosine 1-phosphate; Slc4a4, solute carrier family 4 member 4; CCL, CC chemokine ligand; IL, interleukin; Shh, sonic hedgehog; RA, retinoic acid; PRDX, peroxiredoxin; Cx, connexin; HMGB, high mobility group box; VEGF, vascular endothelial growth factor; TYMP, thymidine phosphorylase; ATP, adenosine triphosphate; ICAM, intercellular adhesion molecule; VCAM, vascular cell adhesion molecule; VE-cadherin, vascular endothelial-cadherin; EphA4, ephrin A4; TNF, tumor necrosis factor; ROS, reactive oxygen species; TREM, triggering receptor of myeloid cells; ZO, zonula occludens; Ang, angiotensin; MIF, migration inhibitory factor; MMP, matrix metalloproteinase; NMDAR, N-methyl-D-aspartate receptor; NMDARE, anti-N-methyl-D-aspartate receptor encephalitis; MS, multiple sclerosis; NMOSD, neuromyelitis optica spectrum disorders; GFAPA, glial fibrillary acidic protein astrocytopathy.

## Data Availability

No new data were created or analyzed in this study.

## References

[1] Kacem K., Lacombe P., Seylaz J., Bonvento G. (1998). Structural organization of the perivascular astrocyte endfeet and their relationship with the endothelial glucose transporter: A confocal microscopy study. Glia.

[2] Bardehle S., Krüger M., Buggenthin F., Schwausch J., Ninkovic J., Clevers H., Snippert H.J., Theis F.J., Meyer-Luehmann M., Bechmann I. (2013). Live imaging of astrocyte responses to acute injury reveals selective juxtavascular proliferation. Nat. Neurosci..

[3] Nielsen S. (1997). Specialized membrane domains for water transport in glial cells: High-resolution immunogold cytochemistry of aquaporin-4 in rat brain. J. Neurosci..

[4] Alvarez J.I., Dodelet-Devillers A., Kebir H., Ifergan I., Fabre P.J., Terouz S., Sabbagh M., Wosik K., Bourbonnière L., Bernard M. (2011). The Hedgehog pathway promotes blood-brain barrier integrity and CNS immune quiescence. Science.

[5] Kubotera H., Ikeshima-Kataoka H., Hatashita Y., Mascaro A.L.A., Pavone F.S., Inoue T. (2019). Astrocytic endfeet re-cover blood vessels after removal by laser ablation. Sci. Rep..

[6] Ma S., Kwon H.J., Huang Z. (2012). A functional requirement for astroglia in promoting blood vessel development in the early postnatal brain. PLoS ONE.

[7] Mills W.A., Woo A.M., Jiang S., Martin J., Surendran D., Bergstresser M., Kimbrough I.F., Eyo U.B., Sofroniew M.V., Sontheimer H. (2022). Astrocyte plasticity in mice ensures continued endfoot coverage of cerebral blood vessels following injury and declines with age. Nat. Commun..

[8] Göbel J., Engelhardt E., Pelzer P., Sakthivelu V., Jahn H.M., Jevtic M., Folz-Donahue K., Kukat C., Schauss A., Frese C.K. (2020). Mitochondria-Endoplasmic Reticulum Contacts in Reactive Astrocytes Promote Vascular Remodeling. Cell Metab..

[9] Brøchner C.B., Holst C.B., Møllgård K. (2015). Outer brain barriers in rat and human development. Front. Neurosci..

[10] Liu H., Wei J.Y., Li Y., Ban M., Sun Q., Wang H.J., Zhao D., Tong P.G., Wang L., Wang K.J. (2023). Endothelial depletion of Atg7 triggers astrocyte–microvascular disassociation at blood–brain barrier. J. Cell Biol..

[11] Lien C.F., Mohanta S.K., Frontczak-Baniewicz M., Swinny J.D., Zablocka B., Górecki D.C. (2012). Absence of glial α-dystrobrevin causes abnormalities of the blood-brain barrier and progressive brain edema. J. Biol. Chem..

[12] Sobue K., Yamamoto N., Yoneda K., E Hodgson M., Yamashiro K., Tsuruoka N., Tsuda T., Katsuya H., Miura Y., Asai K. (1999). Induction of blood-brain barrier properties in immortalized bovine brain endothelial cells by astrocytic factors. Neurosci. Res..

[13] Bell R.D., Winkler E.A., Singh I., Sagare A.P., Deane R., Wu Z., Holtzman D.M., Betsholtz C., Armulik A., Sallstrom J. (2012). Apolipoprotein E controls cerebrovascular integrity via cyclophilin A. Nature.

[14] Garcia J.G., Liu F., Verin A.D., Birukova A., Dechert M.A., Gerthoffer W.T., Bamberg J.R., English D. (2001). Sphingosine 1-phosphate promotes endothelial cell barrier integrity by Edg-dependent cytoskeletal rearrangement. J. Clin. Investig..

[15] Ye Q., Jo J., Wang C.-Y., Oh H., Zhan J., Choy T.J., Kim K.I., D’alessandro A., Reshetnyak Y.K., Jung S.Y. (2024). Astrocytic Slc4a4 regulates blood-brain barrier integrity in healthy and stroke brains via a CCL2-CCR2 pathway and NO dysregulation. Cell Rep..

[16] Liu D., Liao P., Li H., Tong S., Wang B., Lu Y., Gao Y., Huang Y., Zhou H., Shi L. (2024). Regulation of blood-brain barrier integrity by Dmp1-expressing astrocytes through mitochondrial transfer. Sci. Adv..

[17] Jing B., Zhang C., Liu X., Zhou L., Liu J., Yao Y., Yu J., Weng Y., Pan M., Liu J. (2018). Glycosylation of dentin matrix protein 1 is a novel key element for astrocyte maturation and BBB integrity. Protein Cell.

[18] Morales J.E., De A., Miller A.A., Chen Z., McCarty J.H. (2022). Mlc1-Expressing Perivascular Astrocytes Promote Blood-Brain Barrier Integrity. J. Neurosci..

[19] Chen Z., Kelly J.R., Morales J.E., Sun R.C., De A., Burkin D.J., McCarty J.H. (2023). The alpha7 integrin subunit in astrocytes promotes endothelial blood-brain barrier integrity. Development.

[20] Yamasaki R. (2018). Connexins in health and disease. Clin. Exp. Neuroimmunol..

[21] Takeuchi H., Suzumura A. (2014). Gap junctions and hemichannels composed of connexins: Potential therapeutic targets for neurodegenerative diseases. Front. Cell. Neurosci..

[22] Yamamoto T., Ochalski A., Hertzberg E., Nagy J. (1990). LM and EM immunolocalization of the gap junctions protein connexin43 in rat brain. Brain Res..

[23] Yamamoto T., Ochalski A., Hertzberg E.L., Nagy J.I. (1990). On the organization of astrocytic gap junctions in rat brain as suggested by LM and EM immunohistochemistry of connexin43 expression. J. Comp. Neurol..

[24] Nagy J.I., Ochalski P., Li J., Hertzberg E. (1997). Evidence for co-localization of another connexin-43 at astrocytic gap junctions in rat brain. Neuroscience.

[25] Nagy J.I., Patel D., Ochalski P.A.Y., Stelmack G. (1999). Connexin30 in rodent, cat and human brain: Selective expression in gray matter astrocytes, co-localization with connexin30 at gap junctions and late developmental appearance. Neuroscience.

[26] Nagy J.I., Li X., Rempel J., Stelmack G., Patel D., Staines W.A., Yasumura T., Rash J.E. (2001). Connexin26 in adult rodent CNS: Demonstration at astrocytic gap junctions and co-localization with connexin30 and connexin43. J. Comp. Neurol..

[27] Meşe G., Richard G., White T.W. (2007). Gap junctions: Basic structure and function. J. Investig. Dermatol..

[28] Sáez J.C., Retamal M.A., Basilio D., Bukauskas F.F., Bennett M.V. (2005). Connexin-based gap junction hemichannels: Gating mechanisms. Biochim. Biophys. Acta.

[29] Alvarez J.I., Katayama T., Prat A. (2013). Glial influence on the blood brain barrier. Glia.

[30] Sirko S., Behrendt G., Johansson P.A., Tripathi P., Costa M.R., Bek S., Heinrich C., Tiedt S., Colak D., Dichgans M. (2013). Reactive glia in the injured brain acquire stem cell properties in response to sonic hedgehog. Cell Stem Cell.

[31] Mizee M.R., Nijland P.G., van der Pol S.M., Drexhage J.A., van Het Hof B., Mebius R., van der Valk P., van Horssen J., Reijerkerk A., de Vries H.E. (2014). Astrocyte-derived retinoic acid: A novel regulator of blood–brain barrier function in multiple sclerosis. Acta Neuropathol..

[32] Yun H.M., Park K.R., Kim E.C., Hong J.T. (2015). PRDX6 controls multiple sclerosis by suppressing inflammation and blood brain barrier disruption. Oncotarget.

[33] Eilam R., Segal M., Malach R., Sela M., Arnon R., Aharoni R. (2018). Astrocyte disruption of neurovascular communication is linked to cortical damage in an animal model of multiple sclerosis. Glia.

[34] Prineas J.W., Lee S. (2019). Multiple sclerosis: Destruction and regeneration of astrocytes in acute lesions. J. Neuropathol. Exp. Neurol..

[35] Brand-Schieber E. (2005). Connexin43, the major gap junction protein of astrocytes, is down-regulated in inflamed white matter in an animal model of multiple sclerosis. J. Neurosci. Res..

[36] Une H., Yamasaki R., Nagata S., Yamaguchi H., Nakamuta Y., Indiasari U.C., Cui Y., Shinoda K., Masaki K., Götz M. (2021). Brain gray matter astroglia-specific connexin 43 ablation attenuates spinal cord inflammatory demyelination. J. Neuroinflamm.

[37] Yamasaki R. (2023). Connexins Control Glial Inflammation in Various Neurological Diseases. Int. J. Mol. Sci..

[38] Bynoe M.S., Viret C., Yan A., Kim D.-G. (2015). Adenosine receptor signaling: A key to opening the blood-brain door. Fluids Barriers CNS.

[39] Vazana U., Veksler R., Pell G.S., Prager O., Fassler M., Chassidim Y., Roth Y., Shahar H., Zangen A., Raccah R. (2016). Glutamate-mediated blood-brain barrier opening: Implications for neuroprotection and drug delivery. J. Neurosci..

[40] Shi J., Xiao Y., Zhang N., Jiao M., Tang X., Dai C., Wang C., Xu Y., Tan Z., Gong F. (2022). HMGB1 from astrocytes promotes EAE by influencing the immune cell infiltration-associated functions of BMECs in mice. Neurosci. Bull..

[41] Xiao M., Xiao Z.J., Yang B., Lan Z., Fang F. (2020). Blood-brain barrier: More contributor to disruption of central nervous system homeostasis than victim in neurological disorders. Front. Neurol..

[42] Spampinato S.F., Merlo S., Costantino G., Sano Y., Kanda T., Sortino M.A. (2022). Decreased astrocytic CCL2 accounts for BAF-312 effect on PBMCs transendothelial migration through a blood brain barrier in vitro model. J. Neuroimmune Pharmacol..

[43] Kimura A., Takekoshi A., Yoshikura N., Hayashi Y., Shimohata T. (2019). Clinical characteristics of autoimmune GFAP astrocytopathy. J. Neuroimmunol..

[44] Zheng W., Shen P., Yu C., Tang Y., Qian C., Yang C., Gao M., Wu Y., Yu S., Tang W. (2023). Ginsenoside Rh1, a novel casein kinase II subunit alpha (CK2α) inhibitor, retards metastasis via disrupting HHEX/CCL20 signaling cascade involved in tumor cell extravasation across endothelial barrier. Pharmacol. Res..

[45] Chang C.Y., Chen W., Ou Y., Lai C., Hu Y., Wu C., Chang C., Chen C. (2015). Disruption of in vitro endothelial barrier integrity by Japanese encephalitis virus-infected astrocytes. Glia.

[46] Rochfort K.D., Collins L.E., Murphy R.P., Cummins P.M. (2014). Downregulation of blood-brain barrier phenotype by proinflammatory cytokines involves NADPH oxidase-dependent ROS generation: Consequences for interendothelial adherens and tight junctions. PLoS ONE.

[47] Wang Y., Jin S., Sonobe Y., Cheng Y., Horiuchi H., Parajuli B., Kawanokuchi J., Mizuno T., Takeuchi H., Suzumura A. (2014). Interleukin-1β induces blood-brain barrier disruption by downregulating Sonic hedgehog in astrocytes. PLoS ONE.

[48] Abadier M., Jahromi N.H., Alves L.C., Boscacci R., Vestweber D., Barnum S., Deutsch U., Engelhardt B., Lyck R. (2015). Cell surface levels of endothelial ICAM-1 influence the transcellular or paracellular T-cell diapedesis across the blood-brain barrier. Eur. J. Immunol..

[49] Argaw A.T., Asp L., Zhang J., Navrazhina K., Pham T., Mariani J.N., Mahase S., Dutta D.J., Seto J., Kramer E.G. (2012). Astrocyte-derived VEGF-A drives blood-brain barrier disruption in CNS inflammatory disease. J. Clin. Investig..

[50] Chapouly C., Argaw A.T., Horng S., Castro K., Zhang J., Asp L., Loo H., Laitman B.M., Mariani J.N., Farber R.S. (2015). Astrocytic TYMP and VEGFA drive blood-brain barrier opening in inflammatory central nervous system lesions. Brain.

[51] Bennett M.V.L., Garré J.M., Orellana J.A., Bukauskas F.F., Nedergaard M., Giaume C., Sáez J.C. (2012). Connexin and pannexin hemichannels in inflammatory responses of glia and neurons. Brain Res..

[52] Mizee M.R., Wooldrik D., Lakeman K.A.M., Hof B.v.H., Drexhage J.A.R., Geerts D., Bugiani M., Aronica E., Mebius R.E., Prat A. (2013). Retinoic acid induces blood-brain barrier development. J. Neurosci..

[53] Sentilhes L., Michel C., Lecourtois M., Catteau J., Bourgeois P., Laudenbach V., Marret S., Laquerrière A. (2010). Vascular endothelial growth factor and its high-affinity receptor (VEGFR-2) are highly expressed in the human forebrain and cerebellum during development. J. Neuropathol. Exp. Neurol..

[54] da Silva S.M., Campos G.D., Gomes F.C., Stipursky J. (2019). Radial glia-endothelial cells’ bidirectional interactions control vascular maturation and astrocyte differentiation: Impact for blood-brain barrier formation. Curr. Neurovascular Res..

[55] Radonjic N.V., Memi F., Ortega J.A., Glidden N., Zhan H., Zecevic N. (2014). The role of sonic hedgehog in the specification of human cortical progenitors in vitro. Cereb. Cortex.

[56] Cheng N., Brantley D.M., Chen J. (2002). The ephrins and Eph receptors in angiogenesis. Cytokine Growth Factor Rev..

[57] Chen F., Liu Z., Peng W., Gao Z., Ouyang H., Yan T., Ding S., Cai Z., Zhao B., Mao L. (2018). Activation of EphA4 induced by EphrinA1 exacerbates disruption of the blood-brain barrier following cerebral ischemia-reperfusion via the Rho/ROCK signaling pathway. Exp. Ther. Med..

[58] Nishioku T., Matsumoto J., Dohgu S., Sumi N., Miyao K., Takata F., Shuto H., Yamauchi A., Kataoka Y. (2010). Tumor necrosis factor-alpha mediates the blood-brain barrier dysfunction induced by activated microglia in mouse brain microvascular endothelial cells. J. Pharmacol. Sci..

[59] Shigemoto-Mogami Y., Hoshikawa K., Sato K. (2018). Activated microglia disrupt the blood-brain barrier and induce chemokines and cytokines in a rat in vitro model. Front. Cell. Neurosci..

[60] Sumi N., Nishioku T., Takata F., Matsumoto J., Watanabe T., Shuto H., Yamauchi A., Dohgu S., Kataoka Y. (2010). Lipopolysaccharide-activated microglia induce dysfunction of the blood-brain barrier in rat microvascular endothelial cells co-cultured with microglia. Cell Mol. Neurobiol..

[61] Schreibelt G., Kooij G., Reijerkerk A., Doorn R., Gringhuis S.I., Pol S., Weksler B.B., Romero I.A., Couraud P., Piontek J. (2007). Reactive oxygen species alter brain endothelial tight junction dynamics via RhoA, PI3 kinase, and PKB signaling. FASEB J..

[62] Liddelow S.A., Guttenplan K.A., Clarke L.E., Bennett F.C., Bohlen C.J., Schirmer L., Bennett M.L., Münch A.E., Chung W.-S., Peterson T.C. (2017). Neurotoxic reactive astrocytes are induced by activated microglia. Nature.

[63] Chang H. (2023). Elevated blood and cerebrospinal fluid biomarkers of microglial activation and blood–brain barrier disruption in anti-NMDA receptor encephalitis. J. Neuroinflamm..

[64] Gaengel K., Genové G., Armulik A., Betsholtz C. (2009). Endothelial-mural cell signaling in vascular development and angiogenesis. Arterioscler. Thromb. Vasc. Biol..

[65] Stark K., Eckart A., Haidari S., Tirniceriu A., Lorenz M., von Brühl M.-L., Gärtner F., Khandoga A.G., Legate K.R., Pless R. (2013). Capillary and arteriolar pericytes attract innate leukocytes exiting through venules and ‘instruct’ them with pattern-recognition and motility programs. Nat. Immunol..

[66] Li Y. (2023). Myeloid-derived MIF drives RIPK1-mediated cerebromicrovascular endothelial cell death to exacerbate ischemic brain injury. Proc. Natl. Acad. Sci. USA.

[67] Tasaki A., Shimizu F., Sano Y., Fujisawa M., Takahashi T., Haruki H., Abe M., Koga M., Kanda T. (2014). Autocrine MMP-2/9 secretion increases the BBB permeability in neuromyelitis optica. J. Neurol. Neurosurg. Psychiatry.

[68] Chen J.T., Chen T.G., Chang Y.C., Chen C.Y., Chen R.M. (2016). Roles of NMDARs in maintenance of the mouse cerebrovascular endothelial cell-constructed tight junction barrier. Toxicology.

[69] Covo-Calvo A., Ruiz A., Richard C., Blondel S., Cavagna S., Strazielle N., Ghersi-Egea J.F., Giraudon P., Marignier R. (2020). Purified IgG from aquaporin-4 neuromyelitis optica spectrum disorder patients alters blood-brain barrier permeability. PLoS ONE.

[70] Muller Kobold A.C., Van Wijk R.T., Franssen C.F., Molema G., Kallenberg C.G., Tervaert J.W. (1999). In vitro up-regulation of E-selectin and induction of interleukin-6 in endothelial cells by autoantibodies in Wegener’s granulomatosis and microscopic polyangiitis. Clin. Exp. Rheumatol..

[71] Del Papa N., Guidali L., Sironi M., Shoenfeld Y., Mantovani A., Tincani A., Balestrieri G., Radice A., Sinico R.A., Meroni P.L. (1996). Anti-endothelial cell IgG antibodies from patients with Wegener’s granulomatosis bind to human endothelial cells in vitro and induce adhesion molecule expression and cytokine secretion. Arthritis Rheum..

[72] Sun Y., Li N., Zhang J., Liu H., Liu J., Xia X., Sun C., Feng X., Gu J., Du C. (2016). Enolase of streptococcus suis serotype 2 enhances blood-brain barrier permeability by inducing IL-8 release. Inflammation.

[73] Huang F., Mao F., Nong W., Gong Z., Lao D., Huang W. (2024). Inhibiting caveolin-1-related Akt/mTOR signaling pathway protects against N-methyl-D-aspartate receptor activation-mediated dysfunction of blood-brain barrier in vitro. Mol. Neurobiol..

[74] McDermott K.W., Barry D.S., McMahon S.S. (2005). Role of radial glia in cytogenesis, patterning and boundary formation in the developing spinal cord. J. Anat..

[75] Goldshmit Y., Homman-Ludiye J., Bourne J.A. (2014). EphA4 is associated with multiple cell types in the marmoset primary visual cortex throughout the lifespan. Eur. J. Neurosci..

[76] Ma S., Kwon H.J., Johng H., Zang K., Huang Z. (2013). Radial glial neural progenitors regulate nascent brain vascular network stabilization via inhibition of Wnt signaling. PLoS Biol..

[77] Zhang S., Zhao J., Sha W.M., Zhang X.P., Mai J.Y., Bartlett P.F., Hou S.T. (2024). Inhibition of EphA4 reduces vasogenic edema after experimental stroke in mice by protecting the blood-brain barrier integrity. J. Cereb. Blood Flow Metab..

[78] Wang H., Xu Z., Xia Z., Rallo M., Duffy A., Matise M.P. (2021). Inactivation of Hedgehog signal transduction in adult astrocytes results in region-specific blood-brain barrier defects. Proc. Natl. Acad. Sci. USA.

[79] Didier N., Romero I.A., Créminon C., Wijkhuisen A., Grassi J., Mabondzo A. (2003). Secretion of interleukin-1beta by astrocytes mediates endothelin-1 and tumour necrosis factor-alpha effects on human brain microvascular endothelial cell permeability. J. Neurochem..

[80] Marchetti L., Francisco D., Soldati S., Haghayegh Jahromi N., Barcos S., Gruber I., Pareja J.R., Thiriot A., von Andrian U., Deutsch U. (2021). ACKR1 favors transcellular over paracellular T-cell diapedesis across the blood-brain barrier in neuroinflammation in vitro. Eur. J. Immunol..

[81] Almolda B., Villacampa N., Manders P., Hidalgo J., Campbell I.L., González B., Castellano B. (2014). Effects of astrocyte-targeted production of interleukin-6 in the mouse on the host response to nerve injury. Glia.

[82] Korff T., Dandekar G., Pfaff D., Fuller T., Goettsch W., Morawietz H., Schaffner F., Augustin H.G. (2006). Endothelial ephrinB2 is controlled by microenvironmental determinants and associates context-dependently with CD31. Arterioscler. Thromb. Vasc. Biol..

[83] Mondo E., Becker S.C., Kautzman A.G., Schifferer M., Baer C.E., Chen J., Huang E.J., Simons M., Schafer D.P. (2020). A Developmental Analysis of Juxtavascular Microglia Dynamics and Interactions with the Vasculature. J. Neurosci..

[84] Császár E., Lénárt N., Cserép C., Környei Z., Fekete R., Pósfai B., Balázsfi D., Hangya B., Schwarcz A.D., Szabadits E. (2022). Microglia modulate blood flow, neurovascular coupling, and hypoperfusion via purinergic actions. J. Exp. Med..

[85] Bisht K., Okojie K.A., Sharma K., Lentferink D.H., Sun Y.-Y., Chen H.-R., Uweru J.O., Amancherla S., Calcuttawala Z., Campos-Salazar A.B. (2021). Capillary-associated microglia regulate vascular structure and function through PANX1-P2RY12 coupling in mice. Nat. Commun..

[86] Mills S.A., Jobling A.I., Dixon M.A., Bui B.V., Vessey K.A., Phipps J.A., Greferath U., Venables G., Wong V.H.Y., Wong C.H.Y. (2021). Fractalkine-induced microglial vasoregulation occurs within the retina and is altered early in diabetic retinopathy. Proc. Natl. Acad. Sci. USA.

[87] Hartmann D.A., Berthiaume A.-A., Grant R.I., Harrill S.A., Koski T., Tieu T., McDowell K.P., Faino A.V., Kelly A.L., Shih A.Y. (2021). Brain capillary pericytes exert a substantial but slow influence on blood flow. Nat. Neurosci..

[88] Argaw A.T., Gurfein B.T., Zhang Y., Zameer A., John G.R. (2009). VEGF-mediated disruption of endothelial CLN-5 promotes blood-brain barrier breakdown. Proc. Natl. Acad. Sci. USA.

[89] Armulik A., Genové G., Mäe M., Nisancioglu M.H., Wallgard E., Niaudet C., He L., Norlin J., Lindblom P., Strittmatter K. (2010). Pericytes regulate the blood-brain barrier. Nature.

[90] Armulik A., Genové G., Betsholtz C. (2011). Pericytes: Developmental, physiological, and pathological perspectives, problems, and promises. Dev. Cell.

[91] Gundersen G.A., Vindedal G.F., Skare Ø., Nagelhus E.A. (2014). Evidence that pericytes regulate aquaporin-4 polarization in mouse cortical astrocytes. Brain Struct. Funct..

[92] Yamamoto S., Muramatsu M., Azuma E., Ikutani M., Nagai Y., Sagara H., Koo B.-N., Kita S., O’donnell E., Osawa T. (2017). A subset of cerebrovascular pericytes originates from mature macrophages in the very early phase of vascular development in CNS. Sci. Rep..

[93] Hutter-Schmid B., Humpel C. (2018). Primary mouse brain pericytes isolated from transgenic Alzheimer mice spontaneously differentiate into a CD11b+ microglial-like cell type in vitro. Exp. Gerontol..

[94] Klement W., Garbelli R., Zub E., Rossini L., Tassi L., Girard B., Blaquiere M., Bertaso F., Perroy J., de Bock F. (2018). Seizure progression and inflammatory mediators promote pericytosis and pericyte-microglia clustering at the cerebrovasculature. Neurobiol. Dis..

[95] Ozen I., Deierborg T., Miharada K., Padel T., Englund E., Genové G., Paul G. (2014). Brain pericytes acquire a microglial phenotype after stroke. Acta Neuropathol..

[96] Villasenor R., Kuennecke B., Ozmen L., Ammann M., Kugler C., Grüninger F., Loetscher H., Freskgård P.-O., Collin L. (2017). Region-specific permeability of the blood-brain barrier upon pericyte loss. J. Cereb. Blood Flow. Metab..

[97] Chow B.W., Gu C. (2017). Gradual Suppression of Transcytosis Governs Functional Blood-Retinal Barrier Formation. Neuron.

[98] Lecuyer M.A., Kebir H., Prat A. (2016). Glial influences on BBB functions and molecular players in immune cell trafficking. Biochim. Biophys. Acta.

[99] Cannella B. (1995). The adhesion molecule and cytokine profile of multiple sclerosis lesions. Ann. Neurol..

[100] Losy J. (1999). Increased serum levels of soluble PECAM-1 in multiple sclerosis patients with brain gadolinium-enhancing lesions. J. Neuroimmunol..

[101] Losy J. (2013). Is MS an inflammatory or primary degenerative disease?. Neural Transm..

[102] Charabati M., Grasmuck C., Ghannam S., Bourbonnière L., Fournier A.P., Lécuyer M.A., Tastet O., Kebir H., Rébillard R.M., Hoornaert C. (2022). DICAM promotes TH17 lymphocyte trafficking across the blood-brain barrier during autoimmune neuroinflammation. Sci. Transl. Med..

[103] Charabati M., Zandee S., Fournier A.P., Tastet O., Thai K., Zaminpeyma R., Lécuyer M.A., Bourbonnière L., Larouche S., Klement W. (2023). MCAM+ brain endothelial cells contribute to neuroinflammation by recruiting pathogenic CD4+ T lymphocytes. Brain.

[104] Yonezawa T., Ohtsuka A., Yoshitaka T., Hirano S., Nomoto H., Yamamoto K., Ninomiya Y. (2003). Limitrin, a novel immunoglobulin superfamily protein localized to glia limitans formed by astrocyte endfeet. Glia.

[105] Puthenparampil M., Marin A., Zanotelli G., Mauceri V., De Napoli F., Gaggiola M., Miscioscia A., Ponzano M., Bovis F., Perini P. (2024). Blood-brain barrier damage associates with glia-related cytokines in the cerebrospinal fluid of patients with multiple sclerosis. Mult. Scler. Relat. Disord..

[106] Nagata S., Yamasaki R., Takase E.O., Iida K., Watanabe M., Masaki K., Wijering M.H.C., Yamaguchi H., Kira J.-I., Isobe N. (2023). Iguratimod ameliorates the severity of secondary progressive multiple sclerosis in model mice by directly inhibiting IL-6 production and Th17 cell migration via mitigation of glial inflammation. Biology.

[107] Ashley S.L., Pretto C.D., Stier M.T., Kadiyala P., Castro-Jorge L., Hsu T.-H., Doherty R., Carnahan K.E., Castro M.G., Lowenstein P.R. (2017). Matrix metalloproteinase activity in infections by an encephalitic virus, mouse adenovirus type 1. J. Virol..

[108] Szychowski K.A., Gmiński J. (2019). Impact of elastin-derived VGVAPG peptide on bidirectional interaction between peroxisome proliferator-activated receptor gamma (Pparγ) and beta-galactosidase (β-Gal) expression in mouse cortical astrocytes in vitro. Naunyn Schmiedebergs Arch. Pharmacol..

[109] Wójtowicz A.K., Sitarz-Głownia A.M., Wnuk A., Kajta M., Szychowski K.A. (2023). Involvement of the peroxisome proliferator-activated receptor gamma (Pparγ) and matrix metalloproteinases-2 and -9 (Mmp-2 and -9) in the mechanism of action of di(2-ethylhexyl)phthalate (DEHP) in cultured mouse brain astrocytes and neurons. Toxicol. In Vitro.

[110] Szychowski K.A., Skóra B., Wójtowicz A.K. (2022). Elastin-Derived Peptides in the Central Nervous System: Friend or Foe. Cell. Mol. Neurobiol..

[111] Gallo V., Armstrong R.C. (2008). Myelin repair strategies: A cellular view. Curr. Opin. Neurol..

[112] Ballester A., Guijarro A., Bravo B., Hernández J., Murillas R., Gallego M.I., Ballester S. (2022). Hedgehog Signalling Modulates Immune Response and Protects against Experimental Autoimmune Encephalomyelitis. Int. J. Mol. Sci..

[113] Zhang Y.L., Qu Y., Song H.H., Cheng G., Lu F., Cui T.T., Gong Y., Ding X.L., Yang Y., Zhang Q. (2024). Isoliquiritigenin alleviates experimental autoimmune encephalomyelitis by modulating in-flammatory and neuroprotective reactive astrocytes. Biomed. Pharmacother..

[114] Xu N., Bai Y., Han X., Yuan J., Wang L., He Y., Yang L., Wu H., Shi H., Wu X. (2023). Taurochenodeoxycholic acid reduces astrocytic neuroinflammation and alleviates experimental autoimmune encephalomyelitis in mice. Immunobiology.

[115] Masaki K., Suzuki S.O., Matsushita T., Matsuoka T., Imamura S., Yamasaki R., Suzuki M., Suenaga T., Iwaki T., Kira J.-I. (2013). Connexin 43 astrocytopathy linked to rapidly progressive multiple sclerosis and neuromyelitis optica. PLoS ONE.

[116] Markoullis K., Sargiannidou I., Schiza N., Hadjisavvas A., Roncaroli F., Reynolds R., Kleopa K.A. (2012). Gap junction pathology in multiple sclerosis lesions and normal-appearing white matter. Acta Neuropathol..

[117] Takase E.O., Yamasaki R., Nagata S., Watanabe M., Masaki K., Yamaguchi H., Kira J.-I., Takeuchi H., Isobe N. (2024). Astroglial connexin 43 is a novel therapeutic target for chronic multiple sclerosis model. Sci. Rep..

[118] Oh J., Kwon T.W., Choi J.H., Kim Y., Moon S.K., Nah S.Y., Cho I.H. (2024). Ginsenoside-Re inhibits experimental autoimmune encephalomyelitis as a mouse model of multiple sclerosis by downregulating TLR4/MyD88/NF-κB signaling pathways. Phytomedicine.

[119] Gao D., Zheng C.C., Hao J.P., Yang C.C., Hu C.Y. (2023). Icariin ameliorates behavioral deficits and neuropathology in a mouse model of multiple sclerosis. Brain Res..

[120] Hong S., Niu M., Meng D., Li A., Dong Q., Zhang J., Tian X., Lu S., Wang Y. (2022). High-density lipoprotein reduces microglia activation and protects against experimental autoimmune encephalomyelitis in mice. Int. Immunopharmacol..

[121] Liu N., Yu W., Sun M., Li X., Zhang W., Wang M. (2024). Dabrafenib mitigates the neuroinflammation caused by ferroptosis in experimental autoimmune encephalomyelitis by up regulating Axl receptor. Eur. J. Pharmacol..

[122] Ma J., Lu Q., Zhao Y., Wang X., Ding G., Wang Y., Cheng X. (2024). Microglia-astrocyte crosstalk is regulated by Astragalus polysaccharides mediated through suppression of Sema4D-PlexinB2 signaling in experimental autoimmune encephalomyelitis. Brain Res..

[123] Balasa R., Barcutean L., Mosora O., Manu D. (2021). Reviewing the Significance of Blood-Brain Barrier Disruption in Multiple Sclerosis Pathology and Treatment. Int. J. Mol. Sci..

[124] Pyka-Fosciak G., Lis G.J., Litwin J.A. (2020). Effect of natalizumab treatment on metalloproteinases and their inhibitors in a mouse model of multiple sclerosis. J. Physiol. Pharmacol..

[125] Bloomgren G., Richman S., Hotermans C., Subramanyam M., Goelz S., Natarajan A., Lee S., Plavina T., Scanlon J.V., Sandrock A. (2012). Risk of natalizumab-associated progressive multifocal leukoencephalopathy. N. Engl. J. Med..

[126] Ho P.R., Koendgen H., Campbell N., Haddock B., Richman S., Chang I. (2017). Risk of natalizumab-associated progressive multifocal leukoencephalopathy in patients with multiple sclerosis: A retrospective analysis of data from four clinical studies. Lancet Neurol..

[127] Oshima Y., Tanimoto T., Yuji K., Tojo A. (2019). Drug-associated progressive multifocal leukoencephalopathy in multiple sclerosis patients. Mult. Scler..

[128] Major E.O., Yousry T.A., Clifford D.B. (2018). Pathogenesis of progressive multifocal leukoencephalopathy and risks associated with treatments for multiple sclerosis: A decade of lessons learned. Lancet Neurol..

[129] Cortese I., Reich D.S., Nath A. (2021). Progressive multifocal leukoencephalopathy and the spectrum of JC virus-related disease. Nat. Rev. Neurol..

[130] Bernard-Valnet R., Koralnik I.J., Du Pasquier R. (2021). Advances in Treatment of Progressive Multifocal Leukoencephalopathy. Ann. Neurol..

[131] Janosschka C., Lindner M., Koppers N., Starost L., Liebmann M., Eschborn M., Schneider-Hohendorf T., Windener F., Schafflick D., Fleck A.K. (2023). Enhanced pathogenicity of Th17 cells due to natalizumab treatment: Implications for MS disease rebound. Proc. Natl. Acad. Sci. USA.

[132] Sica F., Centonze D., Buttari F. (2019). Fingolimod Immune Effects Beyond Its Sequestration Ability. Neurol. Ther..

[133] Spampinato S.F., Merlo S., Sano Y., Kanda T., Sortino M.A. (2021). Protective effect of the sphingosine-1 phosphate receptor agonist siponimod on disrupted blood brain barrier function. Biochem. Pharmacol..

[134] Spampinato S.F., Obermeier B., Cotleur A., Love A., Takeshita Y., Sano Y., Kanda T., Ransohoff R.M. (2015). Sphingosine 1 Phosphate at the Blood Brain Barrier: Can the Modulation of S1P Receptor 1 Influence the Response of Endothelial Cells and Astrocytes to Inflammatory Stimuli?. PLoS ONE.

[135] Lennon V.A., Wingerchuk D.M., Kryzer T.J., Pittock S.J., Lucchinetti C.F., Fujihara K., Nakashima I., Weinshenker B.G. (2004). A serum autoantibody marker of neuromyelitis optica: Distinction from multiple sclerosis. Lancet.

[136] Lennon V.A., Kryzer T.J., Pittock S.J., Verkman A., Hinson S.R. (2005). IgG marker of optic-spinal multiple sclerosis binds to the aquaporin-4 water channel. J. Exp. Med..

[137] Sabater L., Giralt A., Boronat A., Hankiewicz K., Blanco Y., Llufriu S., Alberch J., Graus F., Saiz A. (2009). Cytotoxic effect of neuromyelitis optica antibody (NMO-IgG) to astrocytes: An in vitro study. J. Neuroimmunol..

[138] Guo Y., Weigand S.D., Popescu B.F., Lennon V.A., Parisi J.E., Pittock S.J., Parks N.E., Clardy S.L., Howe C.L., Lucchinetti C.F. (2017). Pathogenic implications of cerebrospinal fluid barrier pathology in neuromyelitis optica. Acta Neuropathol..

[139] Uchida T., Mori M., Uzawa A., Masuda H., Muto M., Ohtani R., Kuwabara S. (2017). Increased cerebrospinal fluid metalloproteinase-2 and interleukin-6 are associated with albumin quotient in neuromyelitis optica: Their possible role on blood-brain barrier disruption. Mult. Scler..

[140] Wang Y., Zhang J., Chang H., Wang H., Xu W., Cong H., Zhang X., Liu J., Yin L. (2022). NMO-IgG Induce Interleukin-6 Release via Activation of the NF-κB Signaling Pathway in Astrocytes. Neuroscience.

[141] Wang Y., Bian J., Yao M., Du L., Xu Y., Chang H., Cong H., Wei Y., Xu W., Wang H. (2023). Targeting chemoattractant chemokine (C-C motif) ligand 2 derived from astrocytes is a promising therapeutic approach in the treatment of neuromyelitis optica spectrum disorders. Front. Immunol..

[142] Shimizu F., Schaller K.L., Owens G.P., Cotleur A.C., Kellner D., Takeshita Y., Obermeier B., Kryzer T.J., Sano Y., Kanda T. (2017). Glucose-regulated protein 78 autoantibody associates with blood-brain barrier disruption in neuromyelitis optica. Sci. Transl. Med..

[143] Shimizu F., Takeshita Y., Hamamoto Y., Nishihara H., Sano Y., Honda M., Sato R., Maeda T., Takahashi T., Fujikawa S. (2019). GRP 78 antibodies are associated with clinical phenotype in neuromyelitis optica. Ann. Clin. Transl. Neurol..

[144] Gu Y., Zhong M., He L., Li W., Huang Y., Liu J., Chen Y., Xiao Z. (2019). Epidemiology of antibody-positive autoimmune encephalitis in southwest China: A multicenter study. Front. Immunol..

[145] Gable M.S., Sheriff H., Dalmau J., Tilley D.H., Glaser C.A. (2012). The frequency of autoimmune N-methyl-D-aspartate receptor encephalitis surpasses that of individual viral etiologies in young individuals enrolled in the California Encephalitis Project. Clin. Infect. Dis..

[146] Wandinger K.P., Saschenbrecker S., Stoecker W., Dalmau J. (2011). Anti-NMDA-receptor encephalitis: A severe, multistage, treatable disorder presenting with psychosis. J. Neuroimmunol..

[147] Dalmau J., Tüzün E., Wu H.Y., Masjuan J., Rossi J.E., Voloschin A., Baehring J.M., Shimazaki H., Koide R., King D. (2007). araneoplastic anti-N-methyl-D-aspartate receptor encephalitis associated with ovarian teratoma. Ann. Neurol..

[148] Bersier M.G., Pena C., Rodríguez de Lores Arnaiz G. (2008). The expression of NMDA receptor subunits in cerebral cortex and hippocampus is differentially increased by administration of endobain E, a Na^+^, K^+^-ATPase inhibitor. Neurochem. Res..

[149] Hughes E.G., Peng X., Gleichman A.J., Lai M., Zhou L., Tsou R., Parsons T.D., Lynch D.R., Dalmau J., Balice-Gordon R.J. (2010). Cellular and synaptic mechanisms of anti-NMDA receptor encephalitis. J. Neurosci..

[150] Wagnon I., Hélie P., Bardou I., Regnauld C., Lesec L., Leprince J., Naveau M., Delaunay B., Toutirais O., Lemauff B. (2020). Autoimmune encephalitis mediated by B-cell response against N-methyl-d-aspartate receptor. Brain.

[151] Al-Diwani A., Theorell J., Damato V., Bull J., McGlashan N., Green E., Kienzler A.K., Harrison R., Hassanali T., Campo L. (2022). Cervical lymph nodes and ovarian teratomas as germinal centres in NMDA receptor-antibody encephalitis. Brain.

[152] Pan H., Oliveira B., Saher G., Dere E., Tapken D., Mitjans M., Seidel J., Wesolowski J., Wakhloo D., Klein-Schmidt C. (2019). Uncoupling the widespread occurrence of anti-NMDAR1 autoantibodies from neuropsychiatric disease in a novel autoimmune model. Mol. Psychiatry.

[153] Gong X., Wang N., Zhu H., Tang N., Wu K., Meng Q. (2023). Anti-NMDAR antibodies, the blood-brain barrier, and anti-NMDAR encephalitis. Front. Neurol..

[154] Yu Y., Wu Y., Cao X., Li J., Liao X., Wei J., Huang W. (2021). The Clinical Features and Prognosis of Anti-NMDAR Encephalitis Depends on Blood Brain Barrier Integrity. Mult. Scler. Relat. Disord..

[155] Sharp C.D., Hines I., Houghton J., Warren A., Jackson T.H., Jawahar A., Nanda A., Elrod J.W., Long A., Chi A. (2003). Glutamate causes a loss in human cerebral endothelial barrier integrity through activation of NMDA receptor. Am. J. Physiol. Heart Circ. Physiol..

[156] Yu Y., Wu Y., Wei J., Huang F., Mao F., Nong W., Cao X., Huang W. (2022). NMDA mediates disruption of blood-brain barrier permeability via Rho/ROCK signaling pathway. Neurochem. Int..

[157] Shu Y., Peng F., Zhao B., Liu C., Li Q., Li H., Wang Y., Jiang Y., Lu T., Wang Q. (2023). Transfer of patient’s peripheral blood mononuclear cells (PBMCs) disrupts blood-brain barrier and induces anti-NMDAR encephalitis: A study of novel humanized PBMC mouse model. J. Neuroinflammation.

[158] Gong Z., Lao D., Wu Y., Li T., Lv S., Mo X., Huang W. (2023). Inhibiting PI3K/Akt-Signaling pathway improves neurobehavior changes in anti-NMDAR encephalitis mice by ameliorating blood-brain barrier disruption and neuronal damage. Cell. Mol. Neurobiol..

[159] Verkhratsky A., Kirchhoff F. (2007). NMDA Receptors in glia. Neuroscientist.

[160] Skowronska K., Obara-Michlewska M., Zielińska M., Albrecht J. (2019). NMDA Receptors in Astrocytes: In Search for Roles in Neurotransmission and Astrocytic Homeostasis. Int. J. Mol. Sci..

[161] Ismail F.S., Faustmann P.M. (2020). Astrocytes and their potential role in anti-NMDA receptor encephalitis. Med. Hypotheses.

[162] Ariño H., Armangué T., Petit-Pedrol M., Sabater L., Martinez-Hernandez E., Hara M., Lancaster E., Saiz A., Dalmau J., Graus F. (2016). Anti-LGI1-associated cognitive impairment: Presentation and long-term outcome. Neurology.

[163] van Sonderen A., Thijs R.D., Coenders E.C., Jiskoot L.C., Sanchez E., De Bruijn M.A., van Coevorden-Hameete M.H., Wirtz P.W., Schreurs M.W., Sillevis Smitt P.A. (2016). Anti-LGI1 encephalitis: Clinical syndrome and long-term follow-up. Neurology.

[164] Bien C.G., Vincent A., Barnett M.H., Becker A.J., Blümcke I., Graus F., Jellinger K.A., Reuss D.E., Ribalta T., Schlegel J. (2012). Immunopathology of autoantibody-associated encephalitides: Clues for pathogenesis. Brain.

[165] Tröscher A.R., Klang A., French M., Quemada-Garrido L., Kneissl S.M., Bien C.G., Pákozdy Á., Bauer J. (2017). Selective limbic blood-brain barrier breakdown in a feline model of limbic encephalitis with LGI1 antibodies. Front. Immunol..

[166] Qiao S., Li H., Cui C., Zhang C., Wang A., Jiang W., Zhang S. (2024). CSF Findings in Chinese patients with NMDAR, LGI1 and GABABR antibody-associated encephalitis. J. Inflamm. Res..

[167] Jeltsch-David H., Muller S. (2014). Neuropsychiatric systemic lupus erythematosus: Pathogenesis and biomarkers. Nat. Rev. Neurol..

[168] Bertsias G.K., Boumpas D.T. (2010). Pathogenesis, diagnosis and management of neuropsychiatric SLE manifestations. Nat. Rev. Rheumatol..

[169] Stock A.D., Wen J., Putterman C. (2013). Neuropsychiatric lupus, the blood brain barrier, and the TWEAK/Fn14 pathway. Front. Immunol..

[170] Mader S., Brimberg L., Diamond B. (2017). The role of brain-reactive autoantibodies in brain pathology and cognitive iImpairment. Front. Immunol..

[171] Kowal C., DeGiorgio L.A., Nakaoka T., Hetherington H., Huerta P.T., Diamond B., Volpe B.T. (2004). Cognition and immunity; antibody impairs memory. Immunity.

[172] Hirohata S., Arinuma Y., Yanagida T., Yoshio T. (2014). Blood-brain barrier damages and intrathecal synthesis of anti-N-methyl-D-aspartate receptor NR2 antibodies in diffuse psychiatric/neuropsychological syndromes in systemic lupus erythematosus. Arthritis Res. Ther..

[173] Yun Y., Wang X., Xu J., Jin C., Chen J., Wang X., Wang J., Qin L., Yang P. (2023). Pristane induced lupus mice as a model for neuropsychiatric lupus (NPSLE). Behav. Brain Funct..

[174] Nikolopoulos D., Manolakou T., Polissidis A., Filia A., Bertsias G., Koutmani Y., Boumpas D.T. (2023). Microglia activation in the presence of intact blood-brain barrier and disruption of hippocampal neurogenesis via IL-6 and IL-18 mediate early diffuse neuropsychiatric lupus. Ann. Rheum. Dis..

[175] Karla S., Silman A., Akman-Demir G., Bohlega S., Borhani-Haghighi A., Constantinescu C.S., Houman H., Mahr A., Salvarani C., Sfikakis P.P. (2014). Diagnosis and management of Neuro-Behçet’s disease: International consensus recommendations. J. Neurol..

[176] Borhani-Haghighi A., Kardeh B., Banerjee S., Yadollahikhales G., Safari A., Sahraian M.A., Shapiro L. (2020). Neuro-Behcet’s disease: An update on diagnosis, differential diagnoses, and treatment. Mult. Scler. Relat. Disord..

[177] Hirohata S. (2008). Histopathology of central nervous system lesions in Behçet’s disease. J. Neurol. Sci..

[178] Zhan H., Cheng L., Liu Y., Xu H., Feng X., Liu Y., Li H., Li Z., Wang S., Jin H. (2024). Significance of immunoglobulins synthesis with central nervous system involvement in Neuro-Behçet’s disease. Clin. Chim. Acta.

[179] Tobón G.J., Alard J.É., Youinou P., Jamin C. (2009). Are autoantibodies triggering endothelial cell apoptosis really pathogenic?. Autoimmun. Rev..

[180] Chauhan S.K., Tripathy N.K., Nityanand S. (2006). Antigenic targets and pathogenicity of anti-aortic endothelial cell antibodies in Takayasu arteritis. Arthritis Rheum..

[181] Jamin C., Dugué C., Alard J.É., Jousse S., Saraux A., Guillevin L., Piette J.C., Youinou P. (2005). Induction of endothelial cell apoptosis by the binding of anti-endothelial cell antibodies to Hsp60 in vasculitis-associated systemic autoimmune diseases. Arthritis Rheum..

[182] Regent A., Lofek S., Dib H., Bussone G., Tamas N., Federici C., Broussard C., Guillevin L., Mouthon L. (2014). Identification of target antigens of anti-endothelial cell antibodies in patients with anti-neutrophil cytoplasmic antibody-associated vasculitides: A proteomic approach. Clin. Immunol..

[183] Fang B., McKeon A., Hinson S.R., Kryzer T.J., Pittock S.J., Aksamit A.J., Lennon V.A. (2016). Autoimmune Glial Fibrillary Acidic Protein Astrocytopathy: A Novel Meningoencephalomyelitis. JAMA Neurol..

[184] Flanagan E.P., Hinson S.R., Lennon V.A., Fang B., Aksamit A.J., Morris P.P., Basal E., Honorat J.A., Alfugham N.B., Linnoila J.J. (2017). Glial fibrillary acidic protein immunoglobulin G as biomarker of autoimmune astrocytopathy: Analysis of 102 patients. Ann. Neurol..

[185] Iorio R., Damato V., Evoli A., Gessi M., Gaudino S., Di Lazzaro V., Spagni G., A Sluijs J., Hol E.M. (2018). Clinical and immunological characteristics of the spectrum of GFAP autoimmunity: A case series of 22 patients. J. Neurol. Neurosurg. Psychiatry.

[186] Liao H., Chen Q., Zhang M., Chen W. (2022). MRI features and evolution of autoimmune glial fibrillary acidic protein astrocytopathy: A retrospective cross-sectional and longitudinal study. Mult. Scler. Relat. Disord..

[187] Long Y., Liang J., Xu H., Huang Q., Yang J., Gao C., Qiu W., Lin S., Chen X. (2018). Autoimmune glial fibrillary acidic protein astrocytopathy in Chinese patients: A retrospective study. Eur. J. Neurol..

[188] Chen A.Q., Fang Z., Chen X.L., Yang S., Zhou Y.F., Mao L., Xia Y.P., Jin H.J., Li Y.N., You M.F. (2019). Microglia-derived TNF-α mediates endothelial necroptosis aggravating blood brain-barrier disruption after ischemic stroke. Cell Death Dis..

